# Pre-clinical Imaging of Invasive Candidiasis Using ImmunoPET/MR

**DOI:** 10.3389/fmicb.2018.01996

**Published:** 2018-08-23

**Authors:** Hassan O. J. Morad, Anna-Maria Wild, Stefan Wiehr, Genna Davies, Andreas Maurer, Bernd J. Pichler, Christopher R. Thornton

**Affiliations:** ^1^Wolfson Centre for Age-Related Diseases, King’s College London, London, United Kingdom; ^2^Department of Physical Intelligence, Max Planck Institute for Intelligent Systems, Stuttgart, Germany; ^3^Department of Preclinical Imaging and Radiopharmacy, Werner Siemens Imaging Center, Eberhard Karls University of Tübingen, Tübingen, Germany; ^4^ISCA Diagnostics Ltd. and Biosciences, College of Life and Environmental Sciences, University of Exeter, Exeter, United Kingdom

**Keywords:** monoclonal antibody, computed tomography scanning, invasive candidiasis, MRI imaging, positron emission tomography, invasive fungal disease

## Abstract

The human commensal yeast *Candida* is the fourth most common cause of hospital-acquired bloodstream infections, with *Candida albicans* accounting for the majority of the >400,000 life-threatening infections annually. Diagnosis of invasive candidiasis (IC), a disease encompassing candidemia (blood-borne yeast infection) and deep-seated organ infections, is a major challenge since clinical manifestations of the disease are indistinguishable from viral, bacterial and other fungal diseases, and diagnostic tests for biomarkers in the bloodstream such as PCR, ELISA, and pan-fungal β-D-glucan lack either standardization, sensitivity, or specificity. Blood culture remains the gold standard for diagnosis, but test sensitivity is poor and turn-around time slow. Furthermore, cultures can only be obtained when the yeast resides in the bloodstream, with samples recovered from hematogenous infections often yielding negative results. Consequently, there is a pressing need for a diagnostic test that allows the identification of metastatic foci in deep-seated *Candida* infections, without the need for invasive biopsy. Here, we report the development of a highly specific mouse IgG3 monoclonal antibody (MC3) that binds to a putative β-1,2-mannan epitope present in high molecular weight mannoproteins and phospholipomannans on the surface of yeast and hyphal morphotypes of *C. albicans*, and its use as a [^64^Cu]NODAGA-labeled tracer for whole-body pre-clinical imaging of deep-seated *C. albicans* infections using antibody-guided positron emission tomography and magnetic resonance imaging (immunoPET/MRI). When used in a mouse intravenous (i.v.) challenge model that faithfully mimics disseminated *C. albicans* infections in humans, the [^64^Cu]NODAGA-MC3 tracer accurately detects infections of the kidney, the principal site of blood-borne candidiasis in this model. Using a strain of the emerging human pathogen *Candida auris* that reacts with MC3 *in vitro*, but which is non-infective in i.v. challenged mice, we demonstrate the accuracy of the tracer in diagnosing invasive infections *in vivo*. This pre-clinical study demonstrates the principle of using antibody-guided molecular imaging for detection of deep organ infections in IC, without the need for invasive tissue biopsy.

## Introduction

Infectious diseases are a leading cause of death worldwide, with fungi accounting for an estimated two million life-threatening infections each year (Brown et al., 2012). At present, diagnostic tests that can accurately detect and differentiate infectious organisms are lacking, resulting in the incorrect use of antimicrobial drugs, and emergence of antibiotic and antifungal resistance. Non-invasive molecular imaging holds enormous potential for tracking infectious diseases (Santangelo et al., 2015; Rolle et al., 2016; Wiehr et al., 2016; Davies et al., 2017), but conventional radiological imaging modalities such as computed tomography (CT) and magnetic resonance imaging (MRI), while producing high contrast images of all structures within the human body, rely on structural abnormalities in tissues and organs to differentiate between infectious and non-infectious pathologies (Thornton, 2018). In contrast, molecular imaging is able to deliver highly specific and sensitive antibody-guided imaging of viral (Santangelo et al., 2015), bacterial (Wiehr et al., 2016), and fungal diseases (Rolle et al., 2016; Davies et al., 2017) in animal models of disease, with real potential for translation to the clinical setting (Davies et al., 2017).

Invasive candidiasis (IC) is a life-threatening disease caused by the genus *Candida*. The disease, which encompasses both candidemia (bloodstream yeast infection) and deep-seated candidiasis (infection of the tissues beneath mucosal surfaces) (Clancy and Nguyen, 2013), can occur following gastrointestinal surgery, which allows the fungus to penetrate the mucosa (Vincent et al., 2014), while neutropenia, caused by chemotherapy or by immunosuppression of solid-organ or hematological stem cell transplant patients, impairs immune system recognition, and clearance of *Candida* cells leading to disseminated infections (Safdar and Armstrong, 2011). An estimated 400,000 cases of *Candida* bloodstream infection occur globally each year (Brown et al., 2012), making up ∼3% of all nosocomial infections in Europe, and ∼12% in the United States (Schmiedel and Zimmerli, 2016). IC is now the fourth most common bloodstream infection, behind staphylococcal and enterococcal infections, although IC carries much higher rates of mortality (Wisplinghoff et al., 2004; Lewis, 2009).

While non-*albicans* *Candida* species have emerged as pathogens of immuno-compromised individuals over recent years (Chi et al., 2011; Papon et al., 2013; Pfaller et al., 2014), *Candida albicans* remains the most common cause of mucosal and systemic infections and is responsible for up to 70% of cases worldwide (Diekema et al., 2012; Guinea, 2014). Early detection of the pathogen is critical for prompt and effective treatment with antifungal drugs. The current “gold-standard” for detection relies on culture of the fungus from blood, but blood cultures are positive in only 50–70% of cases, are slow to perform, and are rarely positive in patients with deep-seated candidiasis (Clancy and Nguyen, 2013). While non-culture based assays that detect *Candida* nucleic acids, fungal β-D-glucan, and *Candida* mannan antigen (Mn) and anti-mannan antibodies (A-Mn) in patient sera offer potential advantages over culture (Ellis et al., 2009; Avni et al., 2011; Jaijakul et al., 2012), they have their own inherent weaknesses in specificity and sensitivity, and are unable to identify metastatic foci in deep-seated *Candida* infections (Clancy and Nguyen, 2013).

Positron emission tomography and magnetic resonance imaging (PET/MRI) is an immensely powerful tool for diagnosing cancer, but its use in detecting microbial infections is still in its infancy. Despite this, we have recently shown the enormous potential of immunoPET/MRI for imaging of invasive pulmonary aspergillosis (IPA), a lung disease of immuno-compromised humans caused by the ubiquitous air-borne mold *Aspergillus fumigatus* (Rolle et al., 2016; Davies et al., 2017; Thornton, 2018). In the present study, we set out to determine whether a newly developed *Candida*-specific monoclonal antibody (mAb) (MC3), when conjugated to [^64^Cu] using the chelator 2,2′-(7-(1-carboxy-4-((2,5-dioxopyrrolidin-1-yl)oxy)-4-oxobutyl)-1,4,7-triazonane-1,4-diyl) diacetic acid (NODAGA), could be used as a disease-specific tracer in immunoPET/MRI to detect deep-seated *C. albicans* infections *in vivo* following bloodstream infection. We show, using an intravenous (i.v.) challenge model of IC which faithfully mimics disseminated *C. albicans* infection in humans (MacCallum and Odds, 2005; Conti et al., 2014), the accuracy of the [^64^Cu]NODAGA-MC3 tracer in detecting deep organ infections, and demonstrate that antibody-based immunoPET can be used successfully to non-invasively identify this problematic disease *in vivo*.

## Materials and Methods

### Ethics Statement

Hybridoma work described in this study was conducted under a UK Home Office Project Licence and was reviewed by the institution’s Animal Welfare Ethical Review Board (AWERB) for approval. The work was carried out in accordance with The Animals (Scientific Procedures) Act 1986 Directive 2010/63/EU and followed all the Codes of Practice which reinforce this law, including all elements of housing, care, and euthanasia of the animals. All animal molecular imaging work was performed in the Department of Preclinical Imaging and Radiopharmacy (Werner Siemens Imaging Center, Eberhard Karls University of Tübingen, Germany), and was carried out according to protocols approved by the Regierungspräsidium Tübingen (Permit Number: R9/16) as per guidelines from the European Health Law of the Federation of Laboratory Animal Science Associations (FELASA).

### Fungal Cultures

Fungi used for antibody specificity tests were grown on slopes of Sabouraud dextrose agar [SDA; SD broth (S3306, Sigma), agar (MC006, Neogen) 20 g/L], glucose-peptone-yeast extract agar {GPYA; GPY medium [glucose 40 g/L, bacteriological peptone (LP0037, Oxoid) 5 g/L, yeast extract 5 g/L] containing agar 15 g/L}, or potato dextrose agar [PDA; potato dextrose broth (P6685, Sigma), agar 20 g/L]. All media were autoclaved at 121°C for 15 min prior to use and fungi were grown at 26°C under a 16 h fluorescent light regime.

### Preparation of Immunogen and Immunization Regime

*Candida albicans* strain SC5314 was chosen for hybridoma development as it belongs to the predominant clade of closely related *C. albicans* strains that represents almost 40% of all isolates worldwide, as determined by DNA fingerprinting and multi-locus sequence typing (Soll and Pujol, 2003). Three-day-old GPYA Petri dish cultures of *C. albicans* SC5314 grown at 26°C were flooded with 20 mL of sterile Milli-Q water (MQ-H_2_O) and the suspended cells snap frozen in liquid N_2_, lyophilised and placed at −20°C for long-term storage. Immunogen was prepared by re-suspending lyophilised cells in sterile filtered phosphate-buffered saline (PBS; 137 mM NaCl, 2.7 mM KCl, 8 mM Na_2_HPO_4_, and 1.5 mM KH_2_PO_4_, pH 7.2) and 2 mg/mL suspensions heat-inactivated by placing at 55°C for 45 min. The immunogen was stored at −20°C before animal immunisations. For immunisations, four 6-week-old BALB/c white female mice (Charles River) were each given four intra-peritoneal injections (300 μL per injection) of immunogen at 2-week intervals and a single booster injection was given 5 days before fusion.

### Production and Screening of Hybridomas and Determination of Antibody Specificities

Hybridoma cells were produced by the method described elsewhere (Thornton, 2001) and mAb-producing clones identified in ELISA tests by using soluble antigens from the *C. albicans* SC5314 immunogen immobilized to the wells of Nunc F96 Maxisorp microtiter plates (442402, Thermo Fisher Scientific) at 50 μL/well. Positive cell lines were tested for mAb specificities against surface washings from replicate SDA slope cultures of yeast, yeast-like, and filamentous fungi (**Supplementary Table S1**) prepared as described elsewhere (Thornton, 2001). Specificities of *Candida*-specific mAbs were then further tested using antigen solutions prepared from SDA Petri dish culture plates inoculated with cell suspensions of the human pathogenic yeasts *C. albicans* (SC5314), *Candida glabrata* (CBS4962), *Trichosporon asahii* var. *asahii* (CBS5286), or *Cryptococcus neoformans* (CBS7779), either as single species cultures or as mixed species cultures (**Supplementary Figure S1**). After 24 h incubation at 26°C, antigen solutions were prepared by flooding the plates with 10 mL PBS, suspending cells using sterile L-shaped spreaders, and pelleting of cells by centrifugation at 14,500 rpm for 5 min. Protein concentrations, determined spectrophotometrically at 280 nm (Nanodrop; Agilent Technologies), were adjusted to 60 μg/mL and 50 μL volumes were used to coat the wells of microtiter plates, which were incubated overnight at 4°C. Wells were washed three times with PBST (PBS containing 0.05% (v/v) Tween-20), once with PBS and once with dH_2_O before being air-dried at 23°C in a laminar flow hood. The plates were sealed in plastic bags and stored at 4°C in preparation for assay by ELISA.

### Enzyme-Linked Immunosorbent Assay

Wells containing immobilized antigens were incubated for 15 min with 200 μL of PBS containing 1.0% (w/v) bovine serum albumin (BSA; A7906, Sigma) as a blocker. After a 5-min rinse with PBS, wells were incubated with 50 μL of mAb hybridoma tissue culture supernatant (TCS) for 1 h, after which wells were washed three times, for 5 min each, with PBST. Goat anti-mouse polyvalent immunoglobulin (G, A, M) peroxidase conjugate (A0412, Sigma), diluted 1:1000 in PBST containing 0.5% (w/v) BSA, was added to the wells and incubated for a further hour. The plates were washed with PBST as described, given a final 5 min wash with PBS, and bound antibody visualized by incubating wells with 3,3′,5,5′ Tetramethylbenzidine (T2885, Sigma) substrate solution (Thornton, 2001) for 30 min, after which reactions were stopped by the addition of 3 M H_2_SO_4_. Absorbance values were determined at 450 nm using a microplate reader (Tecan GENios, Tecan Austria GmbH). Control wells were incubated with tissue culture medium (TCM) containing 10% (v/v) fetal bovine serum (FBS; FCS-SA, Labtech) only. All incubation steps were performed at 23°C in sealed plastic bags. The threshold for detection of the antigen in ELISA was determined from control means (2× TCM absorbance values) (Sutula et al., 1986). These values were consistently in the range of 0.050–0.100. Consequently, absorbance values >0.100 were considered as positive for the detection of antigen.

### Determination of Ig Class and Sub-Cloning Procedure

The Ig class of mAbs was determined by using antigen-mediated ELISA (Thornton, 2001). Wells of microtiter plates coated with water-soluble antigens from surface washing of *C. albicans* SC5134 were incubated successively with hybridoma TCS for 1 h, followed by goat anti-mouse IgG1, IgG2a, IgG2b, IgG3, IgM, or IgA-specific antiserum (ISO-2, Sigma) diluted 1:3000 in PBST for 30 min, and rabbit anti-goat peroxidase conjugate (A5420, Sigma) diluted 1:1000 for a further 30 min. Bound antibody was visualized with TMB substrate as described. Hybridoma cell lines were sub-cloned three times by limiting dilution, and cell lines were grown in bulk in a non-selective medium, preserved by slowly freezing in FBS/dimethyl sulfoxide (92:8 v/v), and stored in liquid N_2_.

### Epitope Characterization by Heat, Chemical, and Enzymatic Modification

Heat stability of antigens was investigated by placing surface washings of *C. albicans* SC5134 in a boiling water bath. At 10 min intervals over a 60 min period, 1 mL samples were removed, cooled, and centrifuged at 14,500 rpm for 5 min. Fifty microliters volumes of supernatants were immobilized to the wells of microtiter plates for assay by ELISA as described. For periodate oxidation, microtiter wells coated with soluble antigens in surface washings were incubated with 50 μL of sodium meta-periodate solution (20 mM NaIO_4_ in 50 mM sodium acetate buffer, pH 4.5) or acetate buffer only (control) for 24, 4, 3, 2, 1, or 0 h at 4°C in sealed plastic bags. Plates were given four 3 min PBS washes before processing by ELISA as described. For protease digestions, microtiter wells containing immobilized antigens were incubated with 50 μL of a 0.9 mg/mL solution of pronase (protease XIV; P5147, Sigma), trypsin solution (1 mg/mL in MQ-H_2_O), or PBS and MQ-H_2_O only (controls) for 4 h at 37 or 4°C. Plates were given four 3 min rinses with PBS and then assayed by ELISA as described.

### Purified *Candida albicans* Cell Wall Mannan

*Candida albicans* cell wall mannans, purified by the method of Longbottom et al. (1976), were obtained from the National Institute for Biological Standards and Control (catalog no. 76/515). The lyophilised material was re-suspended in MQ-H_2_O to create a 1 mg/mL stock solution, which was stored at −20°C prior to use. A sample of the mannan stock was diluted 1:2 (v:v) with PBS and then double diluted in PBS across the wells of microtiter plates at 50 μL per well. ELISA was then performed as described. For western blotting studies, the mannan stock was diluted in Laemmli buffer (Laemmli, 1970) and denatured by heating at 95°C for 10 min.

### *Candida albicans* Mannan Mutants

*Candida albicans* wild-type strains SC5134 and NGY152, and mannan mutants of NGY152 [Δ*pmr1*, Δ*och1*, Δ*mnn4*, and Δ*mnt1,2* (Hall and Gow, 2013)], were grown on SDA slopes for 3 days and surface washings containing water-soluble antigens prepared using sterile MQ-H_2_O as described. Surface washings were centrifuged for 5 min at 14,500 rpm, and supernatant protein concentrations determined spectrophotometrically at 280 nm were adjusted to 60 μg/mL using PBS. Fifty-μL volumes were used to coat the wells of microtiter plates, which were assayed by ELISA as described. For western blotting, surface washings were diluted in Laemmli buffer, and denatured by heating at 95°C for 10 min.

To investigate shedding of extracellular antigen (**Supplementary Figure S2**), wild-type and mutant strains of *C. albicans* were grown as SDA slope cultures for 3 days, and yeast cell suspensions prepared as described. After washing with sterile MQ-H_2_O, cell suspensions were adjusted to 10^3^ cells/mL, before 1 mL volumes were added to replicate flasks containing 100 mL of autoclaved GPY medium. Cultures were shaken (125 rpm) at 26°C and, after 3 days, the fluids were removed and centrifuged at 14,500 rpm. Fifty-μL samples of supernatants containing 60 μg protein/mL were used to coat the wells of microtiter plates, which were assayed by ELISA as described. For western blotting, culture fluids were diluted in Laemmli buffer and denatured by heating at 95°C for 10 min.

### Polyacrylamide Gel Electrophoresis and Western Blotting

Sodium dodecyl sulphate-polyacrylamide gel electrophoresis (SDS-PAGE) was carried out using 4–20% gradient polyacrylamide gels (161-1159, Bio-Rad) under denaturing conditions. Proteins were separated electrophoretically at 165 V and pre-stained markers (161-0318, Bio-Rad) were used for molecular weight determinations. For western blotting, separated proteins were transferred electrophoretically onto a PVDF membrane (162-0175, Bio-Rad) for 2 h at 75 V, and the membrane was blocked for 16 h at 4°C with PBS containing 1% (w/v) BSA. Blocked membranes were incubated with hybridoma TCS diluted 1:2 (v/v) with PBS containing 0.5% (w/v) BSA (PBSA) for 2 h at 23°C. After washing three times with PBS, membranes were incubated for 1 h with goat anti-mouse IgG (whole molecule) alkaline phosphatase conjugate (A3562, Sigma), diluted 1:15,000 in PBSA. Membranes were washed three times with PBS, once with PBST and bound antibody visualized by incubation in substrate solution (Thornton, 2008). Reactions were stopped by immersing membranes in dH_2_O, and membranes were then air dried between sheets of Whatman filter paper.

### Immunofluorescence

Microscope slides were sterilized by autoclaving and coated with washed SC5314 cells suspended in 1% (w:v) glucose solution for blastospore production, or in 20% (v:v) FBS to induce the formation of hyphae. The slides were incubated in a moist chamber at 26°C for 16 h (blastospore production) or at 37°C for 5 h (hyphal induction). After air-drying, the cells were fixed according to the method described elsewhere (Thornton, 2001). Fixed cells were incubated with hybridoma TCS for 1 h, followed by three 5 min rinses in PBS. Samples were then incubated with goat anti-mouse polyvalent fluorescein isothiocyanate (FITC) conjugate (F1010, Sigma), diluted 1:40 in PBS, for 30 min. They were given three 5-min washes with PBS and mounted in PBS-glycerol mounting medium (F4680, Sigma) before overlaying with coverslips. All incubation steps were performed at 23°C, in a moist environment to prevent evaporation, and slides were stored in the dark, at 4°C, prior to examination using an epifluorescence microscope (Olympus IX81) fitted with 495 nm (excitation) and 518 nm (emission) filters for FITC.

### Purification of mAb MC3

Hybridoma TCS was harvested by centrifugation at 2147 *g* for 40 min at 4°C, followed by filtration through a 0.8 μM cellulose acetate filter (10462240, GE Healthcare Life Sciences, United Kingdom). Culture supernatant was loaded onto a HiTrap Protein A column (17-0402-01, GE Healthcare Life Sciences) using a peristaltic pump P-1 (18-1110-91, GE Healthcare Life Sciences) with a low pulsation flow of 1 mL/min. Columns were equilibrated with 10 mL of PBS, and column-bound antibody was eluted with 5 mL of 0.1 M glycine-HCl buffer (pH 2.5) with a flow rate of 0.5 mL/min. The buffer of the purified antibody was exchanged to PBS using a disposable PD-10 desalting column (17-0851-01, GE Healthcare Life Sciences). Following purification, the antibody was sterile filtered with a 0.24 μm syringe filter (85037-574-44, Sartorius UK Ltd., United Kingdom) and stored at 4°C. Protein concentration was determined using a spectrophotometer, and purity was confirmed by SDS-PAGE and gel staining using Coomassie Brilliant Blue R-250 dye (Thermo Fisher Scientific).

### Conjugation of mAb MC3

Protein A-purified mAb MC3 was buffered with 0.1 M sodium carbonate (pH 9, Chelex-treated) using an Amicon Ultra-15 filter unit (30 kDa MWCO, Merck Millipore, Darmstadt, Germany). Twenty molar equivalents of chelator p-NCS-Bz-NODAGA (Chematech, Dijon, France) were reacted with the antibody for 60 min at room temperature, with subsequent antibody purification via an Amicon Ultra-15 filter using Chelex-treated 0.25 M sodium acetate (pH 6). This protocol typically yields 1–3 chelators per antibody. Elution profile in High Pressure Size Exclusion Chromatography (HPSEC, Phenomenex BioSep SEC-s3000 300 × 4.6 mm, 1.5 mL/min PBS with 50 mM EDTA) was not discernible from the unconjugated antibody.

### PET Tracer Production and Radiolabeling of NODAGA-MC3 Conjugate

^64^Cu was produced at the in-house radiopharmacy of the Department of Preclinical Imaging and Radiopharmacy in Tübingen, using a PETtrace cyclotron (General Electric Medical Systems). ^64^Cu was generated by 12.5 MeV proton irradiation of enriched ^64^Ni metal (35–75 mg; Isoflex, >98% enrichment) electroplated on a platinum/iridium disk via the ^64^Ni(p,n)^64^Cu nuclear reaction as described previously (Alt et al., 2010). In brief, ^64^Cu was separated from the bulk nickel target and other metallic impurities by acid dissolution followed by anion exchange chromatography (AG1 × 8, Bio-Rad) in aqueous hydrochloric acid media (Trace Select, Sigma-Aldrich). The fractions containing the ^64^Cu product were dried under argon at 120°C.

For antibody radiolabeling, the dry ^64^CuCl_2_ was re-dissolved in 0.1 M HCl and the pH was adjusted to 5–6 using 0.5 M ammonium acetate. 1.3 μg of p-NCS-benzyl-NODAGA conjugated MC3 antibody was added per MBq of ^64^Cu and incubated at 42°C for 60 min. Thin layer chromatography (Polygram SIL G/UV254, Macherey-Nagel, Düren, Germany, mobile phase: 0.1 M sodium citrate, pH 5.0) and radio-HPSEC (as described above) were conducted for quality control of the radiolabeled antibodies [radiochemical purity at least 95% (TLC), HPSEC elution profile corresponding to the unmodified antibody].

### Serum Stability of the [^64^Cu]NODAGA-MC3 Tracer

For serum stability tests, the radiolabeled MC3 antibody was incubated with a threefold volume of C57BL/6 serum at 37°C. Samples were taken after 0, 1, 24, and 48 h and immediately analyzed by using Instant Thin Layer Chromatography-Silica Gel (iTLC-SG, Macherey-Nagel, Düren, Germany, mobile phase: 0.1 M sodium citrate pH 5.0) and radio-HPSEC (as described above).

### Molecular Imaging

#### Mouse Strain and i.v. Injections of *Candida* and [^64^Cu]NODAGA-MC3 Tracer

The animal imaging work was carried out using mice of the same age and sex which were injected with the same doses of pathogen and tracer to minimize bias. Eight to 14-week-old female C57BL/6 OlaHsd mice were purchased from Harlan Laboratories GmbH (Venray, Netherlands). The animals were kept under standardized and sterile environmental conditions (20 ± 1°C room temperature, 50 ± 10% relative humidity, 12 h light-dark cycle) and received food and water *ad libitum*. For inoculum preparation, *C. albicans* SC5314 and *C. auris* strain CBS10913 were grown on GPYA as described, and blastospores harvested after 3 days of incubation at 37°C by flushing cultures with sterile PBS. The spores were washed twice by centrifugation at 400 *g* and re-suspension in fresh sterile PBS. The animal infection and imaging protocol (**Supplementary Figure S3A**) included simultaneous PET/MR imaging of *Candida*-infected and non-infected control mice (PBS only) at three consecutive time points [3, 24, and 48 h post-infection (p.i.)]. For administration of inoculum and tracer, animals were anaesthetized briefly with 1.5% isoflurane and 0.8 L/min 100% oxygen, and their lateral tail veins injected with 20 μg of [^64^Cu]NODAGA-labeled MC3 antibody (corresponding to 12–14 MBq for immunoPET imaging), and 100 μL of blastospore suspension (or PBS only), with each mouse receiving a total of 1 × 10^6^ blastospores.

The severity of the infection model is low since the imaging experiments were performed in healthy, immunocompetent mice. The health and general well-being of the mice were assessed daily, and body weights were measured every 24 h. According to the animal care protocol, the health status of the animals was assessed daily and evaluated using a score sheet. The categories for the score sheet included mobility of the animals, flight reaction, grooming, fur condition, and loss of weight in %. The health status of the mice over the course of the experiments showed no weight loss beyond 20% or other signs which would have led to euthanizing of the mouse. No adverse events occurred during the time course of 2 days of infection and no or marginal weight loss was observed in mice due to infection and/or anesthesia.

### Simultaneous PET/MRI and *ex vivo* Bio-Distributions

*In vivo* bio-distributions of the antibody-based PET tracers were assessed using a small animal PET insert (Bruker BioSpin GmbH, Ettlingen, Germany) yielding a spatial resolution of approximately 1.3 mm in the reconstructed images (Wehrl et al., 2013). Static PET scans (10 min) were acquired 3, 24, and 48 h after the injection of *Candida* inoculum and tracer. During PET and MR imaging, the animals were anesthetized with 1.5% isoflurane mixed with 100% oxygen. PET data were acquired in list-mode, histogrammed in one 10 min time frame and reconstructed using a 2D iterative ordered subset expectation maximization (OSEM2D) algorithm. MR imaging was performed on a 7 T, 300 MHz small animal MR tomograph (Bruker Biospin GmbH) for the acquisition of anatomical information. The images were acquired using a T2 fat saturated 3D sequence with a TE/TR of 90.51/1800.000 ms. Additionally, a T1 3D fast low angle shot (FLASH) sequence with a TE/TR of 6.000/30.000 ms was performed. PET images were normalized to each other, subsequently fused to the respective MR images and analyzed using Inveon Research Workplace software (Siemens Preclinical Solutions, Knoxville, TN, United States). Regions of interest (ROIs) were drawn around the respective tissues based on the anatomical information from the MR images. The imaging data were analyzed blinded and cross-checked by two experienced researchers. Absolute quantification of the PET data is expressed as percentage of the injected dose per cubic centimeter (%ID/cc). After the final PET scan at 48 h, all animals were euthanized by cervical dislocation under deep anesthesia and dissected. Organs were removed and radioactivity was quantified with an aliquot of the injected radiotracer in a γ-counter (Wallac 2480 WIZARD 3″, PerkinElmer, Waltham, MA, United States) using an energy window between 350 and 650 keV. The results are expressed as % injected dose per g (%ID/g) of tissue.

### Statistical Analysis

The number of mice used in each group (*N* = 4–5) was determined by power analysis to allow statistical significance at a threshold of 5% and a power of 90%, with the average values differing by 2 SD. For experiments with more than two investigated groups, statistical significances were calculated using one-way analysis of variance (ANOVA) followed by Tukey’s multiple comparison test conducted with Origin 8 software (OriginLab Corporation, Northampton, MA, United States). Data were considered statistically significant at ^∗^*p* < 0.05. Unless otherwise stated, all quantitative data are shown as the mean ± 1 standard deviation (SD).

## Results

### Production of Hybridomas, Isotyping, and mAb Specificity

**FIGURE 1:**
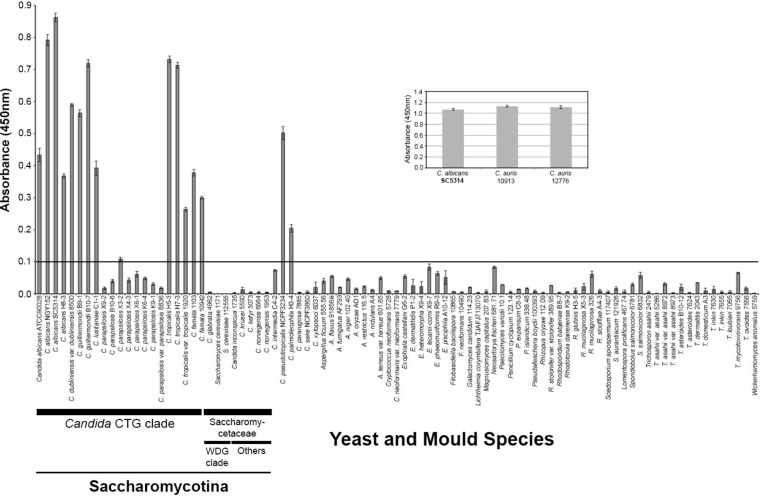
Specificity of mAb MC3 determined by Enzyme-Linked Immunosorbent Assay tests of surface washings containing water-soluble antigens from *Candida* species and related and unrelated yeasts and molds. ELISA absorbance values at 450 nm for soluble antigens from *Candida* species in the CTG clade and Saccharomycetaceae, the emerging human pathogen *Candida auris* (inset), and other related and unrelated yeasts, yeast-like fungi and molds of clinical significance. Wells were coated with 60 μg protein/mL buffer. Bars are the means of three biological replicates ± standard errors and the threshold absorbance value for the detection of antigen is ≥0.1 (indicated by line on graph).

A single fusion was performed and 418 hybridoma cell lines were tested for recognition of the immunogen. The cell line MC3 was selected for further testing based on the strength of its reaction in ELISA and was sub-cloned three times. Isotyping showed that MC3 belonged to immunoglobulin class G3 (IgG3). Specificity of MC3 was determined in ELISA tests by using antigens from a wide range of clinically relevant yeasts, yeast-like fungi, and molds (**Supplementary Table S1**). It reacted with antigens from several *Candida* species, the majority of which belong to the CTG clade (*C. albicans*, *C. dubliniensis*, *C. famata*, *C. guilliermondii*, *C. lusitaniae*, *C. tropicalis*), and also *C. auris*, *C. pseudotropicalis*, and *C. palmioleophila*. It did not react with the other *Candida* species tested, with a wide range of related and unrelated yeasts, yeast-like fungi or molds of clinical relevance (**Figure 1**), or with the human commensal yeast *Saccharomyces cerevisiae*. No cross-reactivity was found with antigens prepared from single or mixed-species cultures of *C. glabrata*, *T. asahii*, or *C. neoformans* (**Supplementary Figure S1**), further demonstrating its specificity for *C. albicans* in polymicrobial cultures.

### Recognition of Purified *Candida albicans* Mannan

**FIGURE 2:**
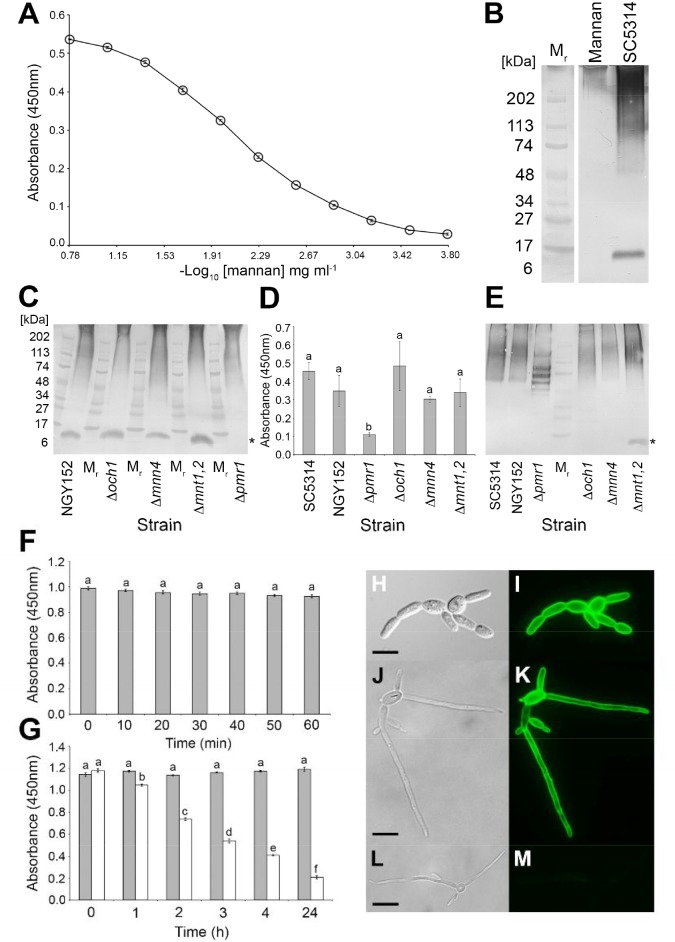
Characterisation of the MC3 antigen, its epitope and spatial distribution in yeast and pseudohyphal cells. **(A)** ELISA absorbance values at 450 nm for purified *C. albicans* cell wall mannan. Each point is the mean of three replicates ± standard errors. **(B)** Western immunoblot with MC3 using purified *C. albicans* cell wall mannan (Mannan) and soluble antigens extracted from immunogen of *C. albicans* SC5314 (SC5314). Wells were loaded with 1.6 μg of protein. M_r_ denotes molecular weight in kDa. **(C)** Western immunoblot with MC3 using soluble antigens in surface washings of the *C. albicans* wild-type strain NGY152 and the mannan mutant strains. Wells were loaded with 1.6 μg of protein. The asterisk (^∗^) indicates the low molecular weight band of ∼12 kDa, assumed to be PLM, which is absent in extracts from the Δpmr1 mutant. **(D)** ELISA absorbance values at 450 nm for culture fluids from the *C. albicans* wild-type strains SC5314 and NGY152 and the mannan mutant strains. Wells were coated with 60 μg protein/mL buffer. Each bar is the mean of three biological replicates ± standard errors and bars with the same letter are not significantly different at *p* < 0.001. **(E)** Corresponding western immunoblot with MC3 using culture fluids from the *C. albicans* wild-type strains SC5134 and NGY152 and mannan mutant strains. Wells were loaded with 1.6 μg of protein. The asterisk (^∗^) indicates the low molecular weight band of ∼12 kDa which is only present in culture fluids of the mutant Δmnt1,2. **(F)** ELISA absorbance values at 450 nm for heat-treated antigens from *C. albicans* SC5314. Wells were coated with 60 μg protein/mL buffer. Each bar is the mean of three biological replicates ± standard errors and bars with the same letter are not significantly different at *p* < 0.001. There was no significant effect of heating on MC3 recognition, indicating that its antigen is heat-stable. **(G)** ELISA absorbance values at 450 nm for periodate-treated antigens (white bars) and control-treated antigens (gray bars) extracted from lyophilised cells of *C. albicans* SC5134. Wells were coated with 60 μg protein/mL buffer. Each bar is the mean of three biological replicates ± standard errors and bars with different letters are significantly different from one another at *p* < 0.001. The significant reduction over time of MC3 recognition of periodate-treated antigens shows that the antibody’s epitope is carbohydrate and contains vicinyl hydroxyl groups. **(H–M)** Photomicrographs of *C. albicans* SC5314 blastospores and hyphae immunostained with MC3 **(I,K)** or TCM control only **(M)** followed by anti-mouse polyvalent Ig fluorescein isothiocyanate conjugate. Bright-field images of *C. albicans* SC5134 blastospores **(H)** and blastospores and hyphae **(J,L)**. **(I–M)** Same fields of view as **(H–L)**, but examined under epifluorescence. Scale bars = 4 μm. Note the intense staining of cell walls of blastospores and hyphae with MC3 **(I,K)**, but lack of staining with TCM only **(M)**, demonstrating specific recognition of *C. albicans* yeast and hyphal cells by the antibody.

Mannan, the major carbohydrate of the *Candida* cell wall, consisting of α-1-6, α-1-2, α-1-3, and β-1-2 structures (Netea et al., 2008; Hall and Gow, 2013), was investigated as the putative antigen bound by MC3. In ELISA tests, MC3 recognized purified *C. albicans* cell wall mannan at concentrations as low as ≥1.3 μg/mL (**Figure 2A**). The mAb also recognized purified mannans with molecular weights >200 kDa (**Figure 2B**). This pattern of binding is consistent with mAbs shown previously to bind to β-1,2-mannan structures (Jouault et al., 1998; Poulain et al., 2002). Western blot binding patterns with soluble antigens extracted from the *C. albicans* SC5314 immunogen showed strong reaction of MC3 with glycosylated antigens with molecular weights between ∼100 and >200 kDa, and a less strong reaction with those of lower molecular weight, between ∼50 and ∼100 kDa (**Figure 2B**). This staining pattern is consistent with mAbs shown to bind to *C. albicans* mannan-containing antigens (Torosantucci et al., 1991). MAb MC3 also reacted with an antigen with a molecular weight of ∼12 kDa (**Figure 2B**), which is consistent with recognition of the cell wall glycolipid antigen phospholipomannan (PLM) (Trinel et al., 1993; Jouault et al., 1998; Poulain et al., 2002; Mille et al., 2012). PLM contains exclusively β-1,2-mannans, which are also found to decorate mannoprotein structures in the *C. albicans* cell wall (Trinel et al., 1999; Mille et al., 2004; Fradin et al., 2008). Taken together, these results suggest that the epitope of MC3 comprises β-1,2-mannan structures.

### *Candida albicans* Mannan Mutants

The characteristics of *C. albicans* mannan mutants have been published elsewhere (Bates et al., 2005; Munro et al., 2005; Hall and Gow, 2013; Shahana et al., 2013), and show that the mutant Δ*och1* lacks branched outer chain N-mannan structures, the mutant Δ*mnn4* lacks phosphomannan on N-branched chains, and mutant Δ*mnt1,2* has truncated O-mannan. The mutant Δ*pmr1* differs to these other mutants in that disruption of the *PMR1* gene results in truncation of all branched N-mannan, O-mannan, and PLM structures (Bates et al., 2005; Munro et al., 2005; Hall and Gow, 2013). In western blotting studies, using soluble antigens in surface washings (**Figure 2C**), MC3 reacted with high molecular weight mannan-containing antigens with a similar binding profile to that of the SC5314 immunogen (**Figure 2B**). This high molecular weight binding pattern was similar for all of the strains tested (**Figure 2C**). MC3 also reacted with the ∼12 kDa antigen, assumed to be PLM, in all strains except Δ*pmr1* (marked with asterisk in **Figure 2C**).

Shedding of antigens by *C. albicans* strains SC5134 and NGY152 was shown to peak after 3 days growth in liquid cultures (**Supplementary Figure S2**). Consequently, samples of 3-day-old culture fluids from wild-type and mutant strains of *C. albicans* were tested for recognition of extracellular antigens by MC3 in ELISA tests (**Figure 2D**). There was no significant difference in MC3 detection of extracellular antigens from the wild-type strains SC5134 and NGY152, and mutants Δ*och1*, Δ*mnn4*, and Δ*mnt1,2*. However, there was a significant reduction in binding of MC3 to extracellular antigens from Δ*pmr1* compared with the other mutants and wild-type strains. Western blotting studies conducted with the same samples (**Figure 2E**) showed that MC3 reacted strongly with high molecular weight extracellular antigens, but the staining pattern with Δ*pmr1* was markedly different to the other strains. Furthermore, the ∼12 kDa antigen present in surface washings of NGY152, Δ*och1*, Δ*mnn4*, and Δ*mnt1,2* (**Figure 2C**) was only present in culture fluids with mutant Δ*mnt1,2* (marked with asterisk in **Figure 2E**).

### Epitope Characterization by Heat, Chemical, and Enzymatic Modification

*Candida albicans* SC5314 antigens were subjected to heat (**Figure 2F**), periodate (**Figure 2G**), and enzymatic (**Supplementary Table S2**) treatments to further elucidate the epitope bound by MC3. Reductions in mAb binding in ELISA following heat treatment would indicate that its epitope is heat labile. However, there was no significant reduction in MC3 binding over 60 min of heating, showing that its epitope is heat stable (**Figure 2F**). Reductions in mAb binding following chemical digestion with periodate shows that its epitope is carbohydrate and contains vicinal hydroxyl groups. Significant reductions in MC3 binding were observed after 1 h of periodate treatment (**Figure 2G**), showing that its epitope consists of carbohydrate moieties. Reductions in mAb binding following treatment with pronase shows that its epitope consists of protein, while reductions with trypsin indicate a protein epitope containing positively charged lysine and arginine side chains. Consequently, the lack of reduction in MC3 binding following digestion of immobilized antigen with pronase or trypsin confirmed that the antibody does not bind to a protein epitope (**Supplementary Table S2**).

### Immunofluorescence

Immunofluorescence studies showed that the MC3 antigen is present on the cell wall surfaces of *C. albicans* blastospores (**Figures 2H–K**), and hyphae (**Figures 2J,K**) of *C. albicans*. The lack of fluorescence of control samples (**Figures 2L,M**) further demonstrates the specificity of mAb MC3 for the different morphotypes of the pathogen.

### PET/MR Imaging of Invasive Candidiasis Using [^64^Cu]NODAGA-MC3

For immunoPET imaging, MC3 was conjugated with the chelator NODAGA. The conjugated antibody was then labeled with the radionuclide ^64^Cu, which allows consecutive imaging for up to 3 days. The specific activity of the [^64^Cu]NODAGA-MC3 tracer used in the *in vivo* bio-distribution studies was determined to be in the region of 620-770 MBq/mg at the time point of injection. Radiochemical purity (TLC) was 95.4%. The serum stability of the [^64^Cu]NODAGA-MC3 tracer was determined by iTLC and radio-HPSEC, with the analysis showing no concerning signs of proteolytic degradation, protein aggregation, or copper transchelation to serum proteins over a 48 h time period (**Supplementary Figure S4**).

**FIGURE 3:**
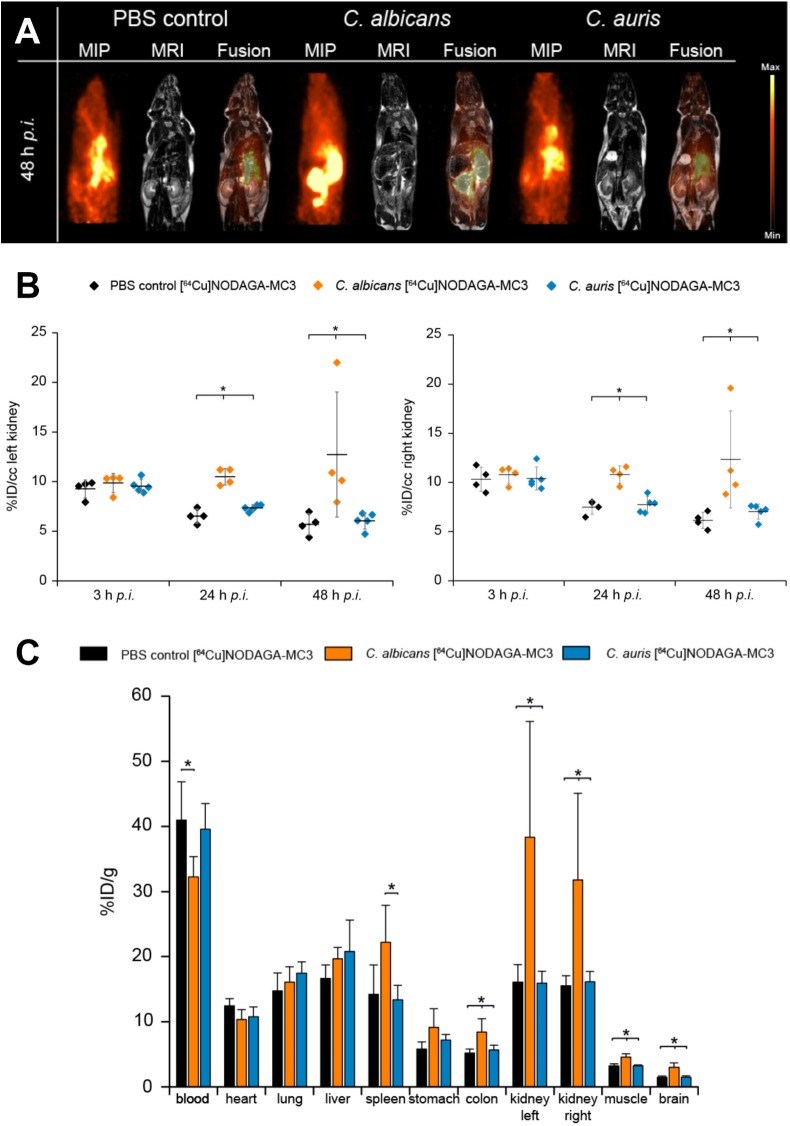
**(A)** *In vivo* biodistribution of [^64^Cu]NODAGA-MC3 in PET/MR imaging at 48 h p.i. Coronal Maximum Intensity Projection (MIP), MR and fused PET/MR images of PBS-treated (control) mice, and *C. albicans*-infected and *C. auris*-infected mice injected with the tracer. The acquired images reveal specific uptake of the tracer in the left and right kidneys of *C. albicans*-infected mice, but not in the kidneys of PBS-treated (control) mice. The specificity of the tracer was further demonstrated using a strain of *C. auris* which, while reactive with MC3 *in vitro* using ELISA (**Figure 1**), was non-infective in the i.v. challenge model. Here, uptake of the tracer in the kidneys was similar to the uptake found in the PBS control mice. **(B)** Quantification of the PET images for the left and right kidneys of the three different groups of mice at 3, 24, and 48 h p.i. Significantly higher uptake of the [^64^Cu]NODAGA-MC3 tracer is seen in the left and right kidneys of *C. albicans*-infected mice compared to PBS-treated (control) and *C. auris*-infected animals at 24 and 48 h p.i. Data are expressed as mean ± SD %ID/cc. Group differences were examined using one-way ANOVA, followed by *post hoc* Tukey–Kramer, with significant differences at ^∗^*p* < 0.05. **(C)** *Ex vivo* biodistribution at 48 h p.i. Significantly higher uptake of the [^64^Cu]NODAGA-MC3 tracer in the left and right kidneys of *C. albicans*-infected mice, compared to the kidneys of corresponding PBS-treated (control) and *C. auris*-infected animals, confirms the PET/MR imaging and *in vivo* biodistribution data at 48 h p.i. In addition to increased uptake of the tracer in the kidneys, significantly higher uptake was also shown in the colons, muscles, and brains of *C. albicans*-infected mice compared to *C. auris*-infected and PBS-treated mice, significantly higher uptake in the spleens of *C. albicans*-infected mice compared to *C. auris*-infected mice, but significantly reduced uptake in the blood of *C. albicans*-infected mice compared to PBS-treated (control) animals. Data are the results of *N* = 4–5 animals per group and expressed as the mean ± SD %ID/g.

Mice were given tail vein injections with blastospores of *C. albicans* or *C. auris* and the [^64^Cu]NODAGA-MC3 tracer, and bio-distributions evaluated by simultaneous PET/MRI at 3, 24, and 48 h post-infection. Representative images are shown in **Supplementary Figures S5A** (3 h), **S5B** (24 h), and **Figure 3A** (48 h), where the same mice are imaged at each time point. Control mice were injected with PBS in place of blastospores, but were otherwise treated similarly. PET imaging showed uptake of the tracer in the left and right kidneys of *C. albicans*-infected animals at 24 h (**Supplementary Figure S5B**) and 48 h (**Figure 3A**) p.i., that was absent in the kidneys of PBS control animals and animals injected with *C. auris*. While there was considerable variability between animals, *in vivo* quantification, expressed as %ID/cc (**Figure 3B**), showed that uptake of the antibody tracer in the left and right kidneys was significantly greater in *C. albicans*-infected animals compared to PBS controls and *C. auris*-infected mice. At 24 h p.i., a significantly higher %ID/cc of the [^64^Cu]NODAGA-MC3 tracer was observed in the left and right kidneys of *C. albicans*-infected mice (10.5 ± 0.8 and 10.8 ± 0.9 %ID/cc, respectively) compared to PBS controls (left kidney: 6.5 ± 0.7 %ID/cc, right kidney: 7.5 ± 0.7 %ID/cc), and *C. auris*-treated animals (left kidney: 7.4 ± 0.3 %ID/cc, right kidney: 7.7 ± 0.8 %ID/cc). The uptake of [^64^Cu]NODAGA-MC3 was found to be highest in the left and right kidneys of *C. albicans*-infected animals 48 h p.i. with 12.7 ± 6.3 and 12.3 ± 4.9 %ID/cc, respectively, compared to the significantly lower uptake of the tracer in the kidneys of PBS treated (left kidney: 5.7 ± 1.1 %ID/cc, right kidney: 6.2 ± 0.8%ID/cc) and *C. auris*-infected (left kidney: 6.1 ± 0.8 %ID/cc, right kidney: 7.0 ± 0.8 %ID/cc) mice. Increased uptake of the tracer was also found in the spleen (**Supplementary Figure S6A**) and brain (**Supplementary Figure S6B**), but not liver (**Supplementary Figure S6C**), of *C. albicans*-infected mice. The uptake of [^64^Cu]NODAGA-MC3 was significantly higher in the brain of *C. albicans*-infected mice 24 and 48 h p.i. with 2.1 ± 0.1 and 1.8 ± 0.2 %ID/cc, respectively, compared to PBS controls (24 h p.i.: 1.7 ± 0.1 %ID/cc, 48 h p.i.: 1.5 ± 0.1 %ID/cc) and *C. auris*-infected animals (24 h p.i.: 1.6 ± 0.1 %ID/cc, 48 h p.i.: 1.4 ± 0.1 %ID/cc). In all three organs, the trend in %ID/cc was a gradual decline over time. Increased uptake of the tracer was also found in the muscle tissues of *C. albicans*-infected mice at 24 h, but this increase was not sustained at 48 h (**Supplementary Figure S6D**). Results from *in vivo* PET imaging were confirmed by the *ex vivo* biodistribution data (%ID/g) at 48 h p.i. (**Figure 3C**), showing significantly higher uptake of [^64^Cu]NODAGA-MC3 in the left and right kidneys of *C. albicans*-infected mice (left kidney: 31.8 ± 13.3 %ID/g, right kidney: 38.4 ± 17.8 %ID/g) compared to the PBS controls (left kidney: 15.5 ± 1.6 %ID/g, right kidney: 16.1 ± 2.7 %ID/g), and *C. auris*-infected animals (left kidney: 16.1 ± 1.6 %ID/g, right kidney: 15.9 ± 1.8 %ID/g). Significant increases in tracer uptake were also seen in the brain (*C. albicans*: 3.0 ± 0.7 %ID/g, *C. auris*: 1.5 ± 0.2 %ID/g, PBS: 1.5 ± 0.2 %ID/g), muscle (*C. albicans*: 4.6 ± 0.5 %ID/g, *C. auris*: 3.2 ± 0.1 %ID/g, PBS: 3.2 ± 0.3 %ID/g), and colon (*C. albicans*: 8.4 ± 2.1 %ID/g, *C. auris*: 5.7 ± 0.7 %ID/g, PBS: 5.2 ± 0.6 %ID/g) of *C. albicans*-infected mice compared to PBS controls and *C. auris*-infected mice, while there was a significant increase in uptake of the tracer in the spleen of *C. albicans*-infected mice (22.2 ± 5.7 %ID/g) compared to the *C. auris* mice (13.4 ± 2.2 %ID/g). There was a significant reduction in the tracer content of blood of *C. albicans*-infected animals (32.3 ± 3.1 %ID/g) compared to the PBS control animals (41.0 ± 5.9 %ID/g).

Significant decreases in body weight after i.v. challenge were found in mice injected with *C. albicans*, but there were no significant decreases in the body weights of PBS control mice and *C. auris*-infected mice over the study period (**Supplementary Figure S7**).

## Discussion

Fungal infections of humans are responsible for at least as many deaths as tuberculosis or malaria, with the most common fungal pathogens (*Aspergillus*, *Candida*, *Cryptococcus*, and *Pneumocystis*) infecting an estimated two million individuals worldwide (Brown et al., 2012). Most of these infections are in immunocompromised or critically ill patients, with *C. albicans* the most common cause (up to 70%) of IC in ICU patients and of recipients of transplanted abdominal organs (Calandra et al., 2016), with an average mortality rate of 40%.

As with all invasive fungal diseases, diagnosis of IC is a major challenge, but prompt and accurate detection is crucial so that appropriate antifungal drug treatment can be started rapidly. In diagnosing IC, three entities must be considered: (1) candidemia without deep-seated candidiasis; (2) candidemia with deep-seated candidiasis; and (3) deep-seated candidiasis without candidemia. Culture of *Candida* from blood samples, considered the gold standard diagnostic test for IC, is positive in only 50–70% of cases, is slow, and is rarely positive in patients with deep-seated candidiasis (Clancy and Nguyen, 2013). Combined determinations of *Candida* mannan (Mn) and anti-Mn (A-Mn) antibodies using the Platelia *Candida* ELISA formats (Ellis et al., 2009) is a useful aid in monitoring patients at high risk for IC but relies on circulating antigen being present when a blood sample is taken. The “pan-fungal” (1→3)-β-D-glucan test is recommended as an indicator of invasive fungal infection (Alexander et al., 2010; Lamoth et al., 2012) but the test lacks specificity to *Candida*, cross-reacting with a number of other yeasts and molds, including *Trichosporon* and *Aspergillus* species, and so its use in detecting IC is limited (Pickering et al., 2005; Karageorgopoulos et al., 2011). Attempts have been made to track *Candida* infections *in vivo*, with Jacobsen et al. (2014) recently reporting bioluminescence imaging for real-time monitoring of IC in mice. While a powerful technique, it relies on bioluminescent *C. albicans* reporter strains expressing firefly luciferase, meaning that optical (bioluminescence) imaging can only be performed using a single genetically modified strain of the fungus, and is in general limited by the low tissue penetration of light. In the clinical setting, [^18^F]FDG PET/CT has been used to visualize IC and *Candida* lung abscesses (Bleeker-Rovers et al., 2005; Avet et al., 2009), but its accuracy in identifying IC or invasive fungal infections in general remains to be fully proven, since the radiopharmaceutical is unable to discriminate between infectious and non-infectious pathologies or to differentiate between bacterial and fungal infections and inflammation (Rolle et al., 2016). Nevertheless, a recent retrospective comparison of [^18^F]FDG PET/CT to conventional CT imaging has shown the utility of [^18^F]FDG PET/CT as a non-invasive procedure for monitoring responsiveness of *Candida* and mold infections to antifungal drugs once the identity of the infecting organisms has been established using conventional diagnostic procedures (Douglas et al., 2018).

In this paper, we set out to improve the diagnostic accuracy of PET-based imaging for invasive *C. albicans* candidiasis by creating and testing a mAb-based tracer for tracking deep-seated *C. albicans* infections *in vivo*. To this end, we report the development and characterisation of a mannan-specific IgG3 mAb, MC3, and its use in antibody-guided positron emission tomography and magnetic resonance (immunoPET/MR) of IC in a pre-clinical mouse model of disease. Recently, we reported the use of immunoPET/MR to image IPA caused by the ubiquitous environmental mold *A. fumigatus* (Rolle et al., 2016; Davies et al., 2017; Thornton, 2018), but this is the first time, to our knowledge, that the potential of immunoPET/MRI to track deep-seated *C. albicans* infections *in vivo* has been demonstrated, with the potential for translation of the technology to the clinical setting and human disease detection.

Monoclonal antibody MC3 was raised against a heat-stable carbohydrate epitope on cell wall mannans of *C. albicans* yeast and hyphal morphotypes. This is crucial from a diagnostic perspective since *C. albicans* is a pleomorphic yeast, which regularly switches morphologies during infection (Calderone and Fonzi, 2001). The absence of the ∼12 kDa immunoreactive antigen in the mutant Δ*pmr1*, which has massively reduced PLM, very little O-mannan and a *N*-glycosylation defect, suggests that the MC3 epitope consists of β-1,2-mannan residues (Trinel et al., 1999; Mille et al., 2004; Fradin et al., 2008). This is consistent with immunodominant (1→2)-β-mannan structures in *C. albicans* PLM bound by the protective β-mannan-specific IgG3 mAb, C3.1 (Han et al., 2000; Johnson et al., 2012).

Specificity tests show that MC3 reacts with mannans from several clinically important *Candida* species, but not with a wide range of related and unrelated human pathogenic yeasts and molds of clinical importance. The mAb was able to discriminate *C. albicans* from the human yeasts *C. glabrata*, *C. neoformans*, and *T. asahii* in polymicrobial cultures, a crucial property given the emerging issue of mixed fungemia in critically ill patients (Jensen et al., 2007; Miceli et al., 2011; Badiye et al., 2012; Davies and Thornton, 2014; Yang et al., 2014). The *Candida* species recognized by MC3 are all reported to cause IC (Morgan et al., 1984; Leung et al., 2002; Hawkins and Baddour, 2003; Pfaller et al., 2006, 2014; Jensen and Arendrup, 2011; Khan et al., 2012; Beyda et al., 2013; Borman et al., 2016). *C. albicans* is the most prevalent cause of IC worldwide and thus of the highest clinical significance (Diekema et al., 2012; Guinea, 2014), but all of the other MC3-reactive species (*C. auris*, *C. dubliniensis*, *C. famata*, *C. guilliermondii*, *C. lusitaniae*, *C. palmioleophila*, *C. pseudotropicalis*, *C. tropicalis*) have emerged as serious human pathogens (Morgan et al., 1984; Leung et al., 2002; Hawkins and Baddour, 2003; Pfaller et al., 2006, 2014; Jensen and Arendrup, 2011; Khan et al., 2012; Beyda et al., 2013; Borman et al., 2016). A weakness of the mAb is its inability to detect *C. glabrata*, *C. krusei*, or *C. parapsilosis*, which are also important causes of IC, especially *C. glabrata* (Chi et al., 2011; Papon et al., 2013; Pfaller et al., 2014). Consequently, while MC3 reacts with a number of clinically relevant *Candida* species, especially *C. albicans*, the most common cause of IC in humans, its inability to detect all pathogenic species means that it would need to be used alongside other diagnostic procedures in the first instance.

Notwithstanding the issue of specificity, we have shown that an anti-mannan mAb, MC3, can be deployed in a disease-specific probe ([^64^Cu]NODAGA-MC3) to detect deep-seated *C. albicans* infections *in vivo*, and have demonstrated the accuracy of the antibody-guided approach to molecular imaging of IC in the most commonly used, well-characterized, and reliable experimental model of disease, the (i.v.) challenge model (MacCallum and Odds, 2005; Conti et al., 2014). We used the clinical isolate *C. albicans* SC5314 (to which mAb MC3 was raised) for the pre-clinical immunoPET/MRI studies as it belongs to the predominant clade of closely related *C. albicans* strains that represents almost 40% of all isolates worldwide (Soll and Pujol, 2003). In addition to a PBS control group, we used, as a *Candida* control, a strain of the emerging human pathogen *Candida auris*, since this pathogen reacts with MC3 in *in vitro* ELISA tests, but is non-infective in the i.v. challenge model. The reasons for this lack of pathogenicity are unclear, but strain-specific differences in pathogenicity are documented in this pathogen (Borman et al., 2016).

Detailed studies of the i.v. challenge model using *C. albicans* SC5314 (MacCallum and Odds, 2005; Conti et al., 2014) have shown the overall course of infection of mice to be elimination of the fungus from blood within 5–10 h, followed by a sudden rapid growth of the fungus in kidney and brain, and progressive elimination of fungus from liver, lung, heart, and spleen. Loss of weight of animals after challenge (cachexia) is a predictor of mortality in the i.v. challenge model (MacCallum and Odds, 2005), as so we used this as a proxy indicator of disease development in infected mice in our study. Only the mice infected with *C. albicans* showed cumulative and significant weight loss following injection of the pathogen and tracer into the bloodstream, signifying the establishment and progression of IC in these animals. Using the two *Candida* strains and a PBS control group, we showed the [^64^Cu]NODAGA-MC3 tracer to be highly accurate in detecting deep-seated infections caused by *C. albicans* SC5314. PET imaging showed sustained uptake of the [^64^Cu]NODAGA-MC3 tracer in the kidneys of *C. albicans*-infected animals at 24 and 48 h, which coincides with exponential growth of the fungus in kidneys in the disease model (MacCallum and Odds, 2005; Conti et al., 2014). *In vivo* and *ex vivo* bio-distributions of the tracer followed a trend similar to that of known disease progression, with sustained uptake of the tracer in the kidneys of *C. albicans*-infected mice at 24 and 48 h p.i., as well as increased uptake in the brain. Uptake of [^64^Cu]NODAGA-MC3 was observed in the liver of all treatment groups, but this decreased over the course of the study coincident with progressive reduction in fungal burden in the i.v. challenge model (MacCallum and Odds, 2005). The *in vivo* bio-distribution data showed a steady accumulation of tracer in the spleen of *C. albicans*-infected animals over the 48 h experimental period, while the *ex vivo* data showed a significant increase in uptake at 48 h compared to the PBS controls and *C. auris*-infected animals. This is consistent with spleen infection, since the spleen is a well-documented site of *C. albicans* infection during IC (Vazquez and Sobel, 2011). The *ex vivo* bio-distribution data similarly showed a significant increase in uptake of the tracer in the colon and muscle of *C. albicans*-infected animals, albeit at markedly lower levels than that of the kidneys. Previous studies using a mouse model of IC demonstrated *in vivo* muscle infection caused by *C. albicans* (Oblack et al., 1980), while infection of the colon by *C. albicans* during IC has been shown both in mouse infection models and in humans (Cole et al., 1996; Praneenararat, 2014). There were no significant differences in uptake of the tracer in the heart, lung, and stomach of the three experimental groups. These organs are known to be non-infected by *C. albicans* in the i.v. challenge model (MacCallum and Odds, 2005). The recurring detection of the pathogen in the blood (candidemia) after 24 h of infection with a high infection dose of *C. albicans* was reported in the i.v. challenge model (MacCallum and Odds, 2005). Here, we found a lower uptake of [^64^Cu]NODAGA-MC3 in the blood of *C. albicans*-infected mice at 48 h p.i. compared to the PBS control and *C. auris*-challenged mice. This phenomenon likely resulted from the significantly higher uptake of the tracer in the kidney, spleen, brain, colon, and muscle tissues of the *C. albicans*-infected mice, thereby reducing the amount of unbound tracer in the bloodstream.

## Conclusion

We have shown that antibody-guided molecular imaging (immunoPET/MRI) can be used to detect disseminated infection caused by *C. albicans*, the most important cause of IC in humans. Using a well-established animal model of infection, which faithfully mimics the disease in humans, we have shown that the [^64^Cu]NODAGA-MC3 tracer provides a highly accurate means of detecting kidney, brain, and spleen infections *in vivo*. As the principal sites of deep organ infection in humans (Vazquez and Sobel, 2011), sensitive and specific detection of *C. albicans* in these organs is of paramount importance for timely diagnosis and treatment. A limitation of our work is the short time period over which sequential monitoring of pathogen distribution could be determined after injection with the [^64^Cu]NODAGA-MC3 tracer (up to 48 h post-injection), and the use of an immunocompetent mouse model of candidiasis. This might explain why we did not detect *Candida* infective endocarditis or hepatosplenic candidiasis (although significantly higher uptake of the tracer was found in the spleens of *C. albicans*-infected mice) which, while relatively rare (Halkin and Ksontini, 2007; Yuan, 2016), are nevertheless important clinical manifestations of disseminated *Candida* disease in humans with associated high mortality rates. Since hepatosplenic candidiasis is most common in immuno-compromised patients (Halkin and Ksontini, 2007), an alternative animal model of candidiasis would need to be used that mimics these diseases in immune-depleted individuals (Conti et al., 2014). Adapting the i.v. challenge model used here might allow the accuracy of the tracer in detecting a previously established infection to be determined. Such a strategy is shown in **Supplementary Figure S3B** where, rather than simultaneous injection of blastospores and tracer, the fungal inoculum is injected 24 h prior to tracer injection to allow a systemic infection to first be established. However, we opted to administer the pathogen and tracer simultaneously, as we believed this would minimize stress to the animals, while at the same time allowing the potential of antibody-guiding imaging of IC to be investigated.

In order to develop an antibody-guided tracer that specifically detects all *Candida* species of clinical importance, a mAb would need to be developed that binds to an antigenic determinant common to all *Candida* species, but which is not conserved in other pathogenic yeasts and molds. Furthermore, such an epitope would need to be absent in humans to prevent non-target binding of the mAb to human structures. Our approach to IC detection is essentially translatable to the clinical setting, since the mAb, MC3, binds to a fungal carbohydrate (mannan) and epitope (a putative β-1,2-mannan) which are not found in humans (Barreto-Bertger and Figueirredo, 2014), thereby reducing substantially the likelihood of adverse events using a humanized version of the antibody tracer. The antibody and its mannan target therefore represent excellent candidates for non-invasive detection of IC in humans caused by *C. albicans*, the most common cause of IC in humans. Translation of the mAb-based tracer to the clinical setting will require humanisation of the mouse antibody using CDR grafting technology similar to that recently used by our group for humanisation of the *Aspergillus*-specific mAb JF5 for diagnosis of IPA (Davies et al., 2017).

## Author Contributions

HM, GD, A-MW, BP, SW, and CT conceived and designed the study, and wrote the manuscript. HM, GD, A-MW, BP, SW, AM, and CT developed the methodology.

## Conflict of Interest Statement

BP receives grant/research support from Bayer Healthcare, Boehringer-Ingelheim, and Siemens; however, none of the grants are directly related to this work. CT is director of ISCA Diagnostics Ltd. The remaining authors declare that the research was conducted in the absence of any commercial or financial relationships that could be construed as a potential conflict of interest. The reviewer DM and handling Editor declared their shared affiliation at the time of review.
