# Supplementary material for: Pre-clinical Imaging of Invasive Candidiasis Using ImmunoPET/MR
Source: Front Microbiol. 2018 Aug 23;9:1996. doi: 10.3389/fmicb.2018.01996 (PMC6115526; doi:10.3389/fmicb.2018.01996)
Supplement: Supplementary file 1 [file Data_Sheet_1.docx]

**TABLES**

### Table S1. Details of fungal cultures used in MC3 specificity tests.

| Organism | Isolate Number^a^ | Source^b^ |
| --- | --- | --- |
| *Candida albicans* | ATCC90028 | ATCC |
| *Candida albicans* | NGY152 | CBS |
| *Candida albicans* | SC5314 | SB |
| *Candida albicans* | H8-3 (KT876577) | CRT |
| *Candida auris* | 10913 | CBS |
| *Candida auris* | 12776 | CBS |
| *Candida dubiniensis* var. *dubliniensis* | 8500 | CBS |
| *Candida guilliermondii* | B9-1 (KT876707) | CRT |
| *Candida guilliermondii* | B10-7 (KT876711) | CRT |
| *Candida lusitaniae* | C1-1 (KT876708) | CRT |
| *Candida parapsilosis* | X9-2 (KT876496) | CRT |
| *Candida parapsilosis* | B10-6 (KT876703) | CRT |
| *Candida parapsilosis* | X3-2 (KT876498) | CRT |
| *Candida parapsilosis* | X4-3 (KT876508) | CRT |
| *Candida parapsilosis* | X6-1 (KT876499) | CRT |
| *Candida parapsilosis* | K6-4 (KT876524) | CRT |
| *Candida parapsilosis* | K9-1 (KT876525) | CRT |
| *Candida parapsilosis* var. *parapsilosis* | 8836 | CBS |
| *Candida tropicalis* | H5-3 (KT876574) | CRT |
| *Candida tropicalis* | H7-3 (KT876575) | CRT |
| *Candida tropicalis* var. *tropicalis* | 1920 | CBS |
| *Candida famata* | 1103 | CBS |
| *Candida famata* | 10942 | CBS |
| *Candida glabrata* | 4962 | CBS |
| *Candida inconspicua* | 1735 | CBS |
| *Candida krusei* | 5590 | CBS |
| *Candida kefyr* | 3073 | CBS |
| *Candida norvegensis* | 6564 | CBS |
| *Candida norvegensis* | 1953 | CBS |
| *Candida intermedia* | C4-2 (KT876709) | CRT |
| *Candida palmioleophila* | H3-4 (KT876573) | CRT |
| *Candida pseudotropicalis* | NCPF3234 | NCPF |
| *Candida pararugosa* | 7885 | CBS |
| *Candida sake* | NCPF3860 | NCPF |
| *Candida xylopsoci* | 6037 | CBS |
| *Aspergillus ficuum* | 555.65 | CBS |
| *Aspergillus flavus* | 91856iii | IMI |
| *Aspergillus fumigatus* | AF293 | SK |
| *Aspergillus niger* | 102.40 | CBS |
| *Aspergillus oryzae* | AO1 | CRT |
| *Aspergillus restrictus* | 116.5 | CBS |
| *Aspergillus nidulans* | A4 | FGSC |
| *Aspergillus terreus* var. *terreus* | 601.65 | CBS |
| *Cryptococcus neoformans* | 5728 | CBS |
| *Cryptococcus neoformans* var. *neoformans* | 7779 | CBS |
| *Exophiala castellanii* | G6-2 (KT876528) | CRT |
| *Exophiala dermatitidis* | P1-2 (KT876582) | CRT |
| *Exophiala heteromorpha* | X9-4 (KT876503) | CRT |
| *Exophiala lecanii-corni* | X9-7 (KT876502) | CRT |
| *Exophiala phaeomuriformis* | R9-3 (KT876555) | CRT |
| *Exophiala pisciphila* | A10-2 (KT876616) | CRT |
| *Filobasidiella baccillispora* | 10865 | CBS |
| *Filobasidiella neoformans* | 10490 | CBS |
| *Galactomyces candidum* | 114.23 | CBS |
| *Kluyveromyces marxianus* | 3073 | CBS |
| *Lichtheimia corymbifera* | (TJAFJ713070) | CRT |
| *Lomentospora prolificans* | 467.74 | CBS |
| *Magnusiomyces capitatus* | 207.83 | CBS |
| *Neosartorya fischeri* var. *fischeri* | 681.71 | CBS |
| *Paecilomyces variotii* | 10.1 | CRT |
| *Penicillium cyclopium* | 123.14 | CBS |
| *Penicillium expansum* | C8-3 (KT876716) | CRT |
| *Penicillium islandicum* | 338.48 | CBS |
| *Pseudallescheria boydii* | 100393 | CBS |
| *Rhizopus oryzae* | 112.09 | CBS |
| *Rhizopus stolonifer* var. *stolonifer* | 389.95 | CBS |
| *Rhodosporidium babjenae* | B8-7 (KT876706) | CRT |
| *Rhodotorula dairenensis* | K9-2 (KT876526) | CRT |
| *Rhodotorula glutinis* | H3-5 (KT876598) | CRT |
| *Rhodotorula mucilaginosa* | X5-3 (KT876501) | CBS |
| *Rhodotorula mucilaginosa* | 326 | CBS |
| *Rhodotorula slooffiae* | A4-3 (KT876704) | CRT |
| *Saccharomyces cerevisiae* | 1171 | CBS |
| *Saccharomyces cerevisiae* | 112555 | CBS |
| *Scedosporium apiospermum* | 117407 | CBS |
| *Scedosporium aurantiacum* | 121926 | CBS |
| *Sporidiobolus salmonicolor* | 6781 | CBS |
| *Sporidiobolus salmonicolor* | 6832 | CBS |
| *Trichosporon asahii* | 2479 | CBS |
| *Trichosporon asahii* var. *asahii* | 5286 | CBS |
| *Trichosporon asahii* var. *asahii* | 8972 | CBS |
| *Trichosporon asahii* var. *asahii* | 8973 | CBS |
| *Trichosporon asteroides* | 7624 | CBS |
| *Trichosporon asteroides* | B10-12 (KT876713) | CRT |
| *Trichosporon dermatitis* | 2043 | CBS |
| *Trichosporon domesticum* | A3-1 | CRT |
| *Trichosporon inkin* | 7630 | CBS |
| *Trichosporon inkin* | 7655 | CBS |
| *Trichosporon loubieri* | 7065 | CBS |
| *Trichosporon mycotoxinivorans* | 9756 | CBS |
| *Trichosporon ovoides* | 7556 | CBS |
| *Wickerhamomyces anomalus* | 5759 | CBS |

^a^ CBS; Centraalbureau voor Schimmelcultures, Utrecht, The Netherlands. SK; S. Krappman, Institute of Microbiology and Genetics, Department of Molecular Microbiology and Genetics, Georg-August University, Gottingen, Germany. IMI; International Mycological Institute, Egham, England. FGSC; Fungal Genetics Stock Centre, University of Missouri, Kansas City. CRT; C.R. Thornton, University of Exeter, UK. SB; S. Bates, University of Exeter, UK. NCBI accession numbers of strains are shown in parentheses.

Table S2. Absorbance values at 450 nm for protease-treated antigen in ELISA tests with MC3.

|  | Absorbance (450 nm)^a^ | | | |
| --- | --- | --- | --- | --- |
| Temp (°C) | **Trypsin** | **H_2_O** | **Pronase** | **PBS** |
| 4 | 1.366 ± 0.018 | 1.457 ± 0.010 | 1.313 ± 0.014 | 1.438 ± 0.013 |
| 37 | 1.347 ± 0.009 | 1.474 ± 0.014 | 1.313 ± 0.011 | 1.402 ± 0.028 |

^a^ Each value is the mean of three replicates ± standard error. Values within treatments are not significantly different to one another at p<0.05 (Student’s t-test).

**Supplementary figures and legends**

**
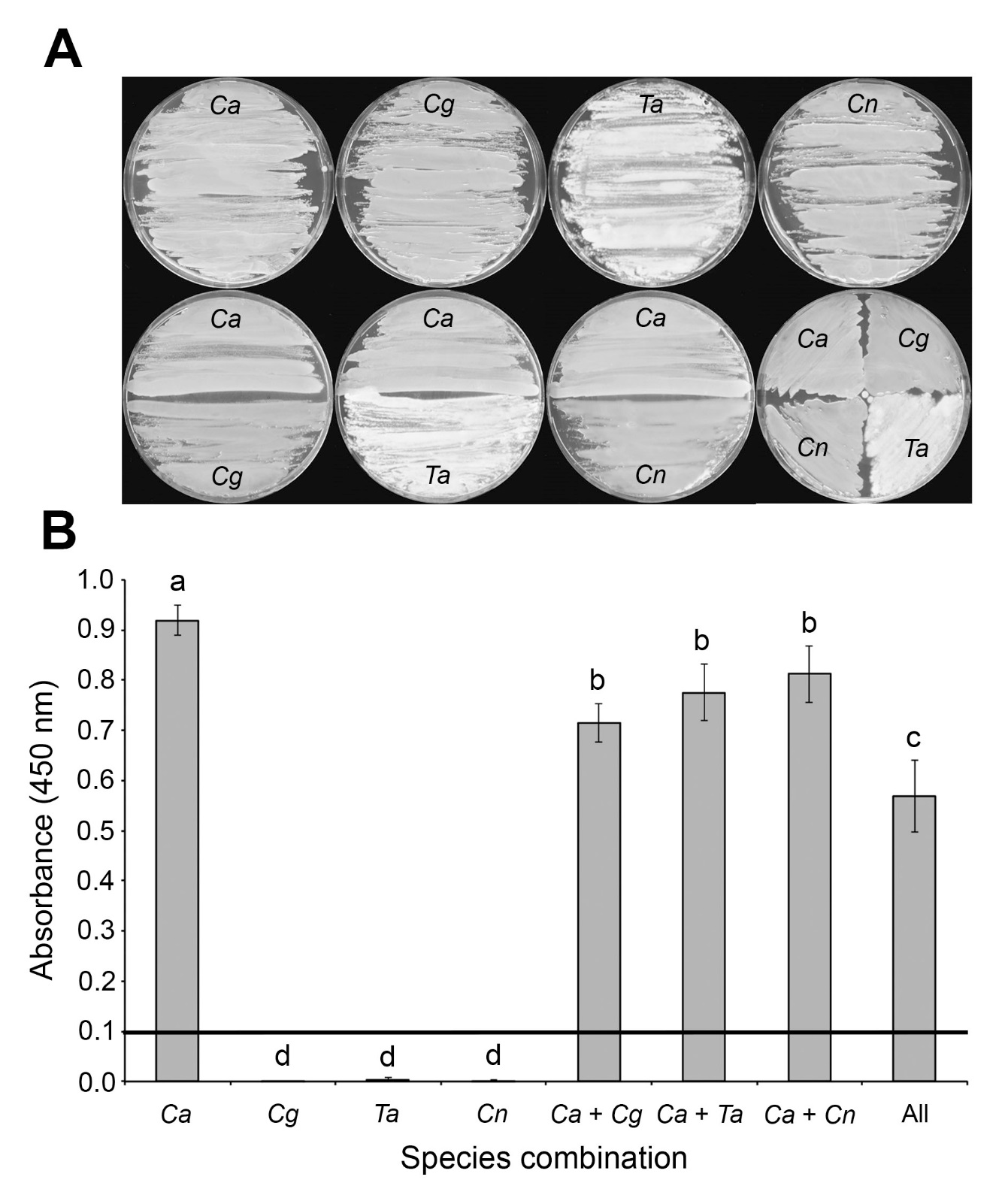
**

**Supplementary Figure 1.** Growth of yeasts as single or mixed species cultures and ELISA tests of soluble antigens using MC3. (A) Sabouraud dextrose agar plates inoculated with different combinations of species: *Candida albicans* SC5314 (Ca), *Candida glabrata* CBS4962 (Cg), *Trichosporon ashaii* var. *asahii* CBS5286 (Ta) and *Cryptococcus neoformans* var. *neoformans* CBS7779 (Cn). (B) Absorbance at 450 nm for antigen solutions tested in ELISA using MC3. Bars are the means of three biological replicates ± standard errors and the threshold absorbance value for the detection of antigen is ≥0.1 (indicated by line on graph). Bars with the same letter are not significantly different at p<0.001 (ANOVA and Tukey-Kramer test).

**
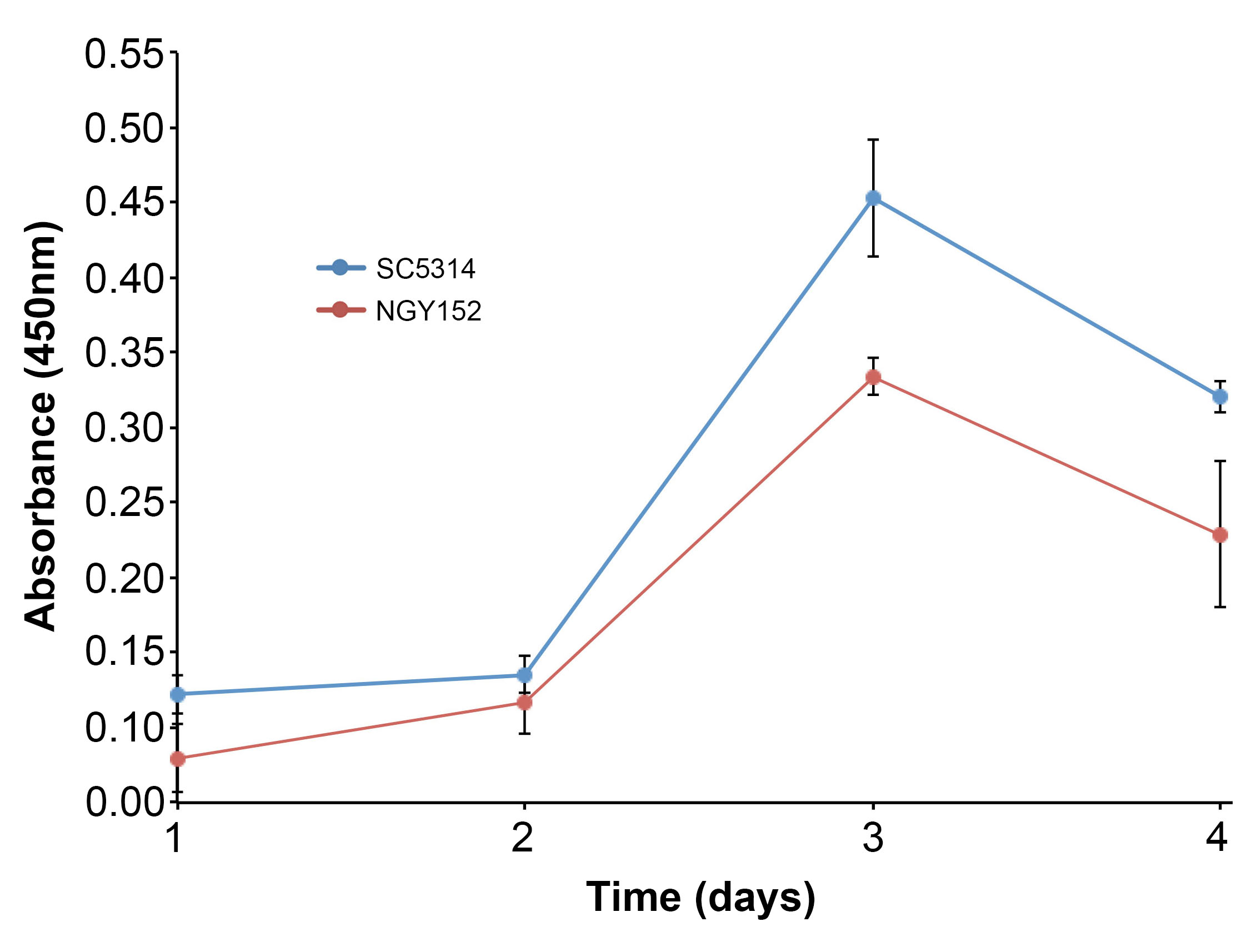
**

**Supplementary Figure 2.** Enzyme-Linked Immunosorbent Assay (ELISA) tests of antigens shed by *C. albicans* strains SC5134 and NGY152 during growth in liquid culture. Washed blastospores were used to inoculate replicate flasks containing liquid GPY medium, and cultures shaken (125 rpm) at 26°C. At 24 h intervals, fluids were collected, centrifuged at 14,500 rpm, and 50 µL samples (diluted in PBS to contain 60 µg protein/mL) used to coat the wells of microtiter plates for assay by ELISA. ELISA absorbance values increased up to 72 h post inoculation concomitant with increased shedding of the MC3 antigen into culture fluids.

**
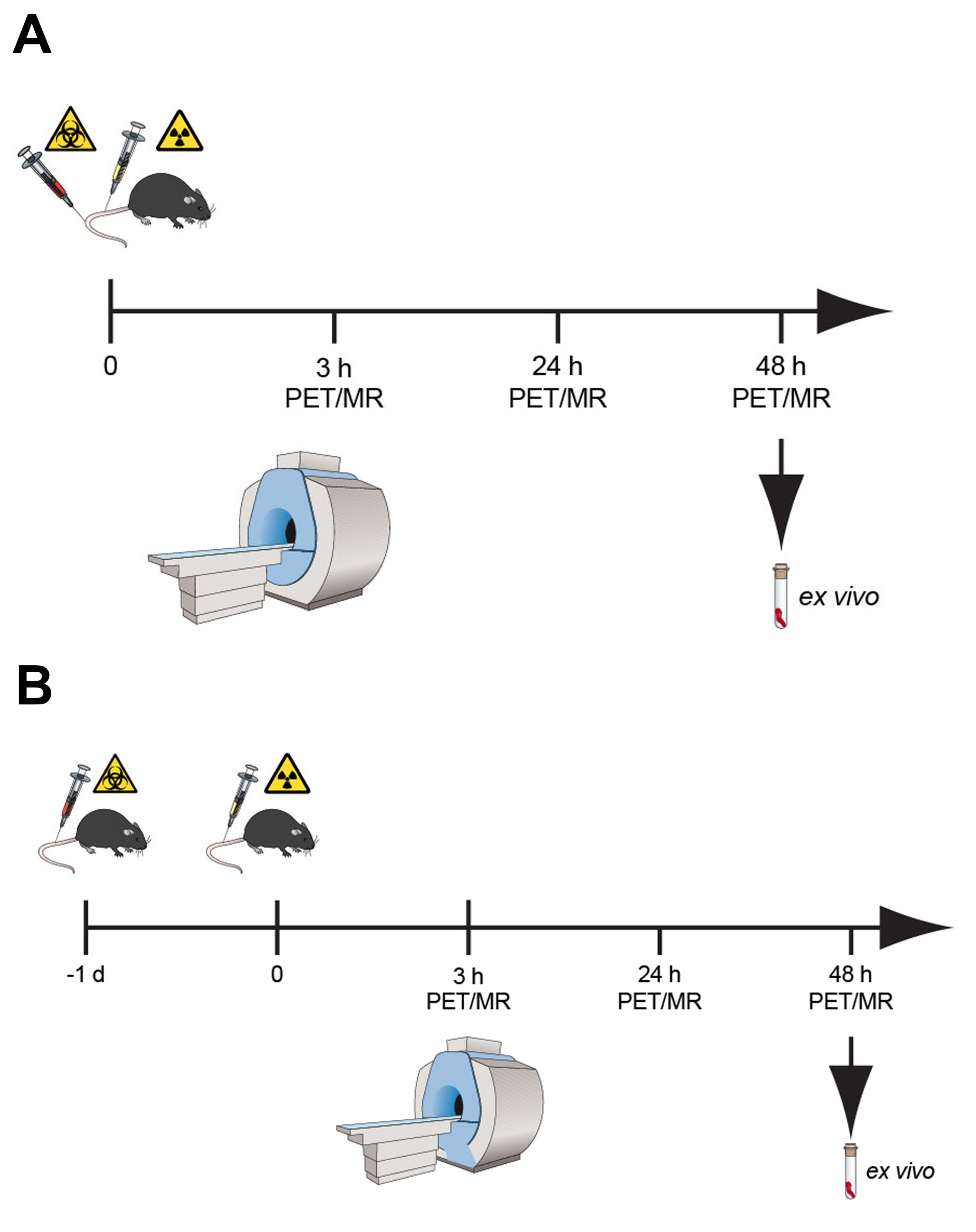
**

**Supplementary Figure 3.** **A.** Schematic representation of the imaging procedure. C57BL/6 mice are injected simultaneously with 20 μg of tracer and 10^6^ *C. albicans* or *C. auris* blastospores/mouse. Concurrent PET/MR imaging of the animals is then performed 3 h, 24 h and 48 h after infection/tracer injection. *Ex vivo* biodistributions are conducted after the last PET scan at 48 h post infection and tracer injection. **B.** Alternative procedure to allow establishment of infection prior to injection of the imaging tracer. Twenty-four h prior to tracer injection, C57BL/6 mice are injected with 10^6^ blastospores/mouse. Twenty-four h later, the animals are injected with 20 μg of tracer, and concurrent PET/MR imaging of the animals is then performed at 3 h, 24 h and 48 h. *Ex vivo* biodistributions are conducted after the last PET scan at 48 h post tracer injection.

**
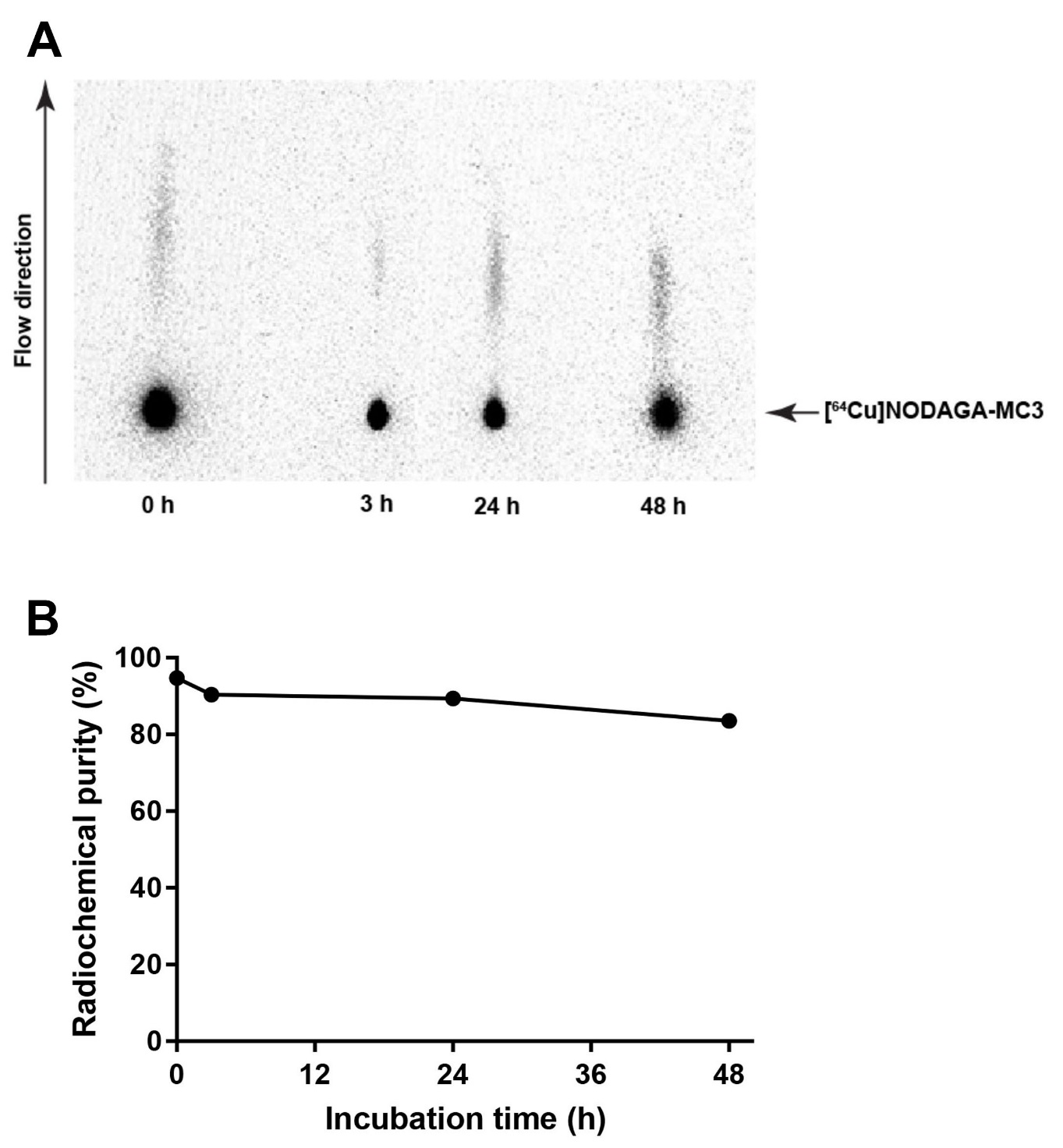
**

**Supplementary Figure 4. A.** Serum stability of the ^64^Cu-labeled, chelator-conjugated MC3 antibody determined by TLC. Sample was run on iTLC-SG paper and then analysed by autoradiography. Arrow = direction of flow. **B.** Quantification of iTLC autoradiography. The results demonstrate high stability of the radiotracer in murine serum.


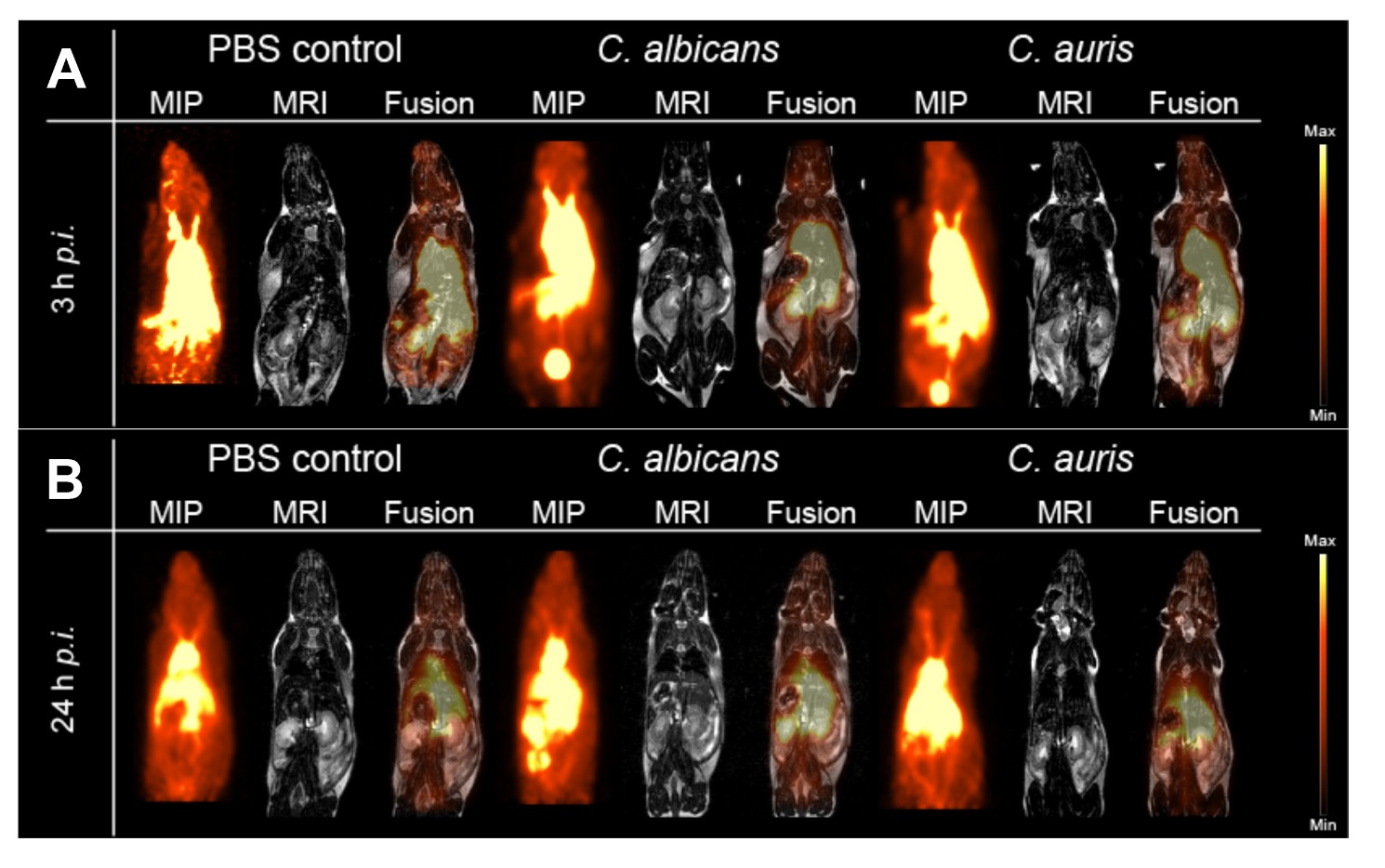


**Supplementary Figure 5. A.** *In vivo* biodistribution of [^64^Cu]NODAGA-MC3 in PET/MR imaging at 3 h *p.i.* Coronal Maximum Intensity Projection (MIP), MR and fused PET/MR images of PBS-treated (control) mice, and *C. albicans*-infected and *C. auris-*infected mice injected with the tracer. **B.** *In vivo* biodistribution of [^64^Cu]NODAGA-MC3 in PET/MR imaging at 24 h *p.i.* Coronal MIP, MR and fused PET/MR images of PBS-treated (control) mice, and *C. albicans*-infected and *C. auris*-infected mice injected with the tracer. The acquired images at 24 h *p.i.* reveal specific uptake of the tracer in the left and right kidneys of *C. albicans*-infected mice, but not in the kidneys of PBS-treated (control) mice. The specificity of the tracer was further demonstrated using a strain of *C. auris* which, while reactive with MC3 *in vitro* using ELISA (Fig. 1), was non-infective in the *i.v.* challenge model. Here, uptake of the tracer in the kidneys was similar to the uptake found in the PBS control mice.


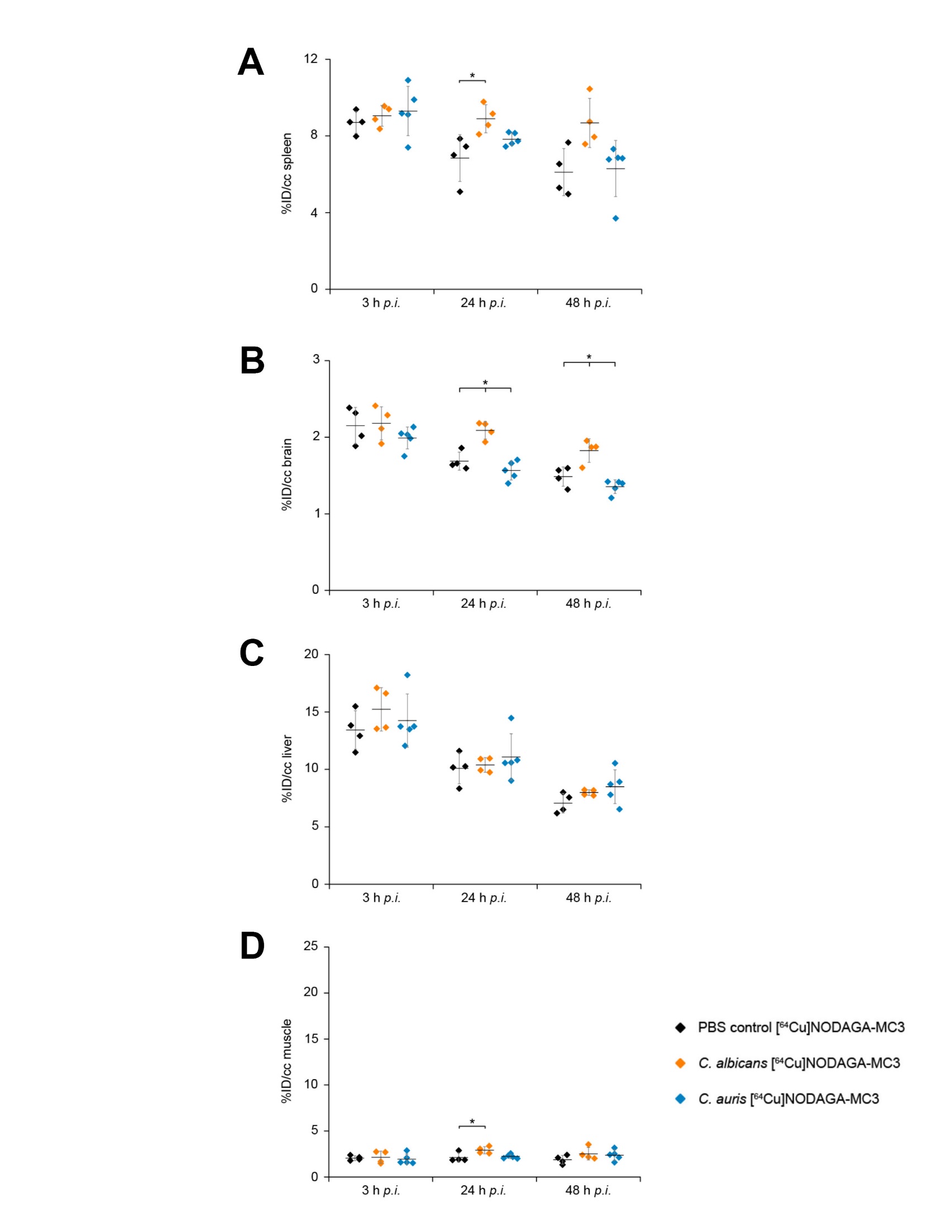


**Supplementary Figure 6.** Quantification of the *in vivo* PET insert data for (A) spleen, (B) brain, (C) liver, and (D) muscle tissues at 3, 24 and 48 h *p.i.* The graphs display the uptake of the [^64^Cu]NODAGA-MC3 tracer in *C. albicans*-infected, *C auris*-infected, and PBS-treated (control) animals in groups of *N* = 4-5 mice. Data are expressed as the mean ± SD %ID/cc. Group differences were examined using one-way ANOVA followed by post hoc Tukey–Kramer, **P* < 0.05.

**
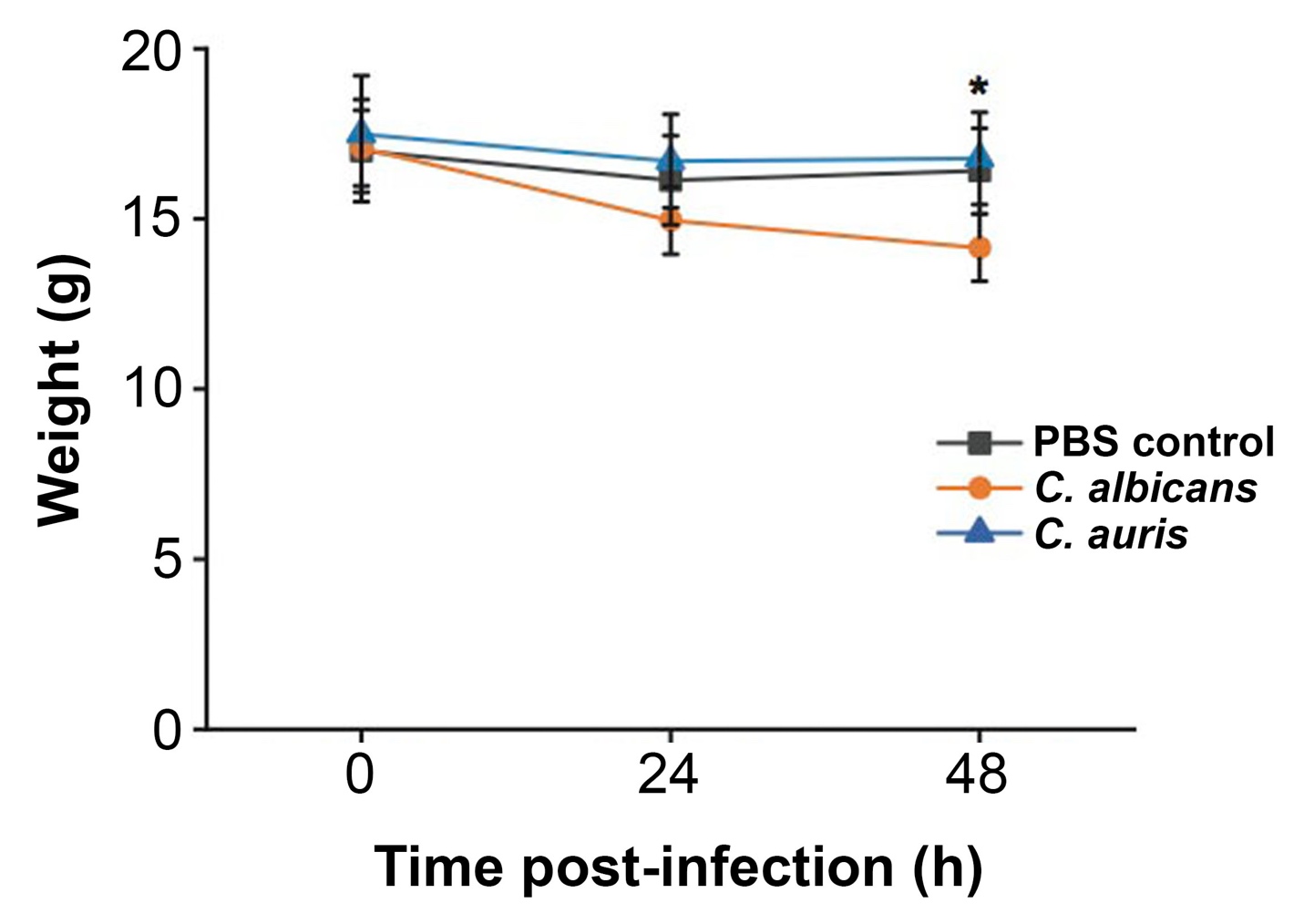
**

**Supplementary Figure 7.** Weights of PBS-treated (control), *C. albicans*-infected, and *C. auris*-infected mice over the 2-day experimental period. Data are expressed as the mean weights ± SD for *N* = 4-5 animals. Group differences at 48 h post-infection were examined using one-way ANOVA followed by post hoc Tukey–Kramer, **P* < 0.05.
